# Adult-Onset Still's Disease: A Case Report

**DOI:** 10.7759/cureus.21033

**Published:** 2022-01-08

**Authors:** Iram Shad, Muhammad Shafique, Syeda A Waris, Farwa Shabbir, Attiya Begum

**Affiliations:** 1 Internal Medicine, Holy Family Hospital, Rawalpindi, PAK; 2 Obstetrics and Gynaecology, District Headquarters Hospital, Rawalpindi, PAK

**Keywords:** negative serological markers, steroids, yamaguchi criteria, autoinflammation, adult onset stills disease

## Abstract

Adult-onset Still's disease (AOSD) is a disorder that is occasionally seen. Autoinflammation is a generally accepted pathogenic mechanism leading to systemic signs and symptoms. We report the case of a young female presenting with high-grade fever, rash, and arthralgias. After a thorough assessment, the diagnosis of adult-onset Still's disease was made based on presenting symptoms and elevated serum ferritin, c-reactive protein (CRP), and absence of serologic markers and confirmed based on Yamaguchi criteria. She was treated with corticosteroids and achieved complete clinical remission.

## Introduction

Adult-onset Still's disease (AOSD), with an incidence of one and 34 cases per million, is among one of the few unconventional causes of pyrexia of unknown origin [1]. The disease usually goes misdiagnosed, as its clinical features are indistinguishable from other causes of febrile illness. The most widely accepted pathogenic mechanism for this disorder is autoinflammation, in which there is an activation of the innate immune system against itself in response to danger signals [2]. There are no specific biomarkers that support the diagnosis. Since AOSD is a diagnosis of exclusion, labeling the patient with this disease requires a lot of work-up to rule out other differentials that appear alike, including infections, malignancy, and other rheumatic diseases [3]. We describe here a case of a young female patient of AOSD who has now achieved remission.

## Case presentation

A 12-year-old girl presented to the medical department of a tertiary care hospital with a complaint of fever for 14 days. Fever was sudden in onset, up to 104°F, continuous, associated with rigors and chills, and partially relieved by antipyretics. Other symptoms included nausea, pain in the oligoarticular joints, maculopapular rash on the chest, and significant weight loss in the past few months. She had no history of prior hospitalization and was fully vaccinated according to the extended program immunization schedule. Physical examination was significant for the patient being lean and lethargic, with a fever and maculopapular salmon-colored rash on the chest with cervical lymphadenopathy.

Laboratory studies were significant for a markedly raised WBC count of 21.9x10^9, platelets 506x10^3, and mild anemia with hemoglobin of 8.8 g/dl. Tests of liver and renal function, prothrombin time (PT), and activated partial thromboplastin time (APTT) were normal. Keeping infective etiologies like urinary tract infection, infective endocarditis, tuberculosis, and brucellosis on top of the list of differential diagnoses, we went for a routine urine examination, urine and blood cultures, echocardiography, brucella antigens, and a chest X-ray, all of which came normal.

We then started workup to rule out malignant causes for which blood peripheral film with retics was sent, which showed neutrophilic leukocytosis and normal retics. Ultrasound examination of the abdomen and neck showed borderline splenomegaly and multiple enlarged cervical lymph nodes, more on the left side, with the largest measuring 13x5.7mm in size. Fine-needle aspiration cytology was done, which showed reactive hyperplasia. Next up, in order to rule out autoimmune causes, rheumatoid factor (RA), antinuclear antibody (ANA) levels, and erythrocyte sedimentation rate (ESR) were sent, all of which came normal with results of (<8IU/mL), (<0.50), and (22 mm/1hr), respectively. A list of common differentials that were ruled out is shown in Table 1.

**Table 1:** Differential diagnoses that were ruled out FNAC: fine-needle aspiration cytology; ANA: antinuclear antibodies; RA: rheumatoid factor; SLE: systemic lupus erythematosus

Infections	
Urinary tract infection	Normal urine routine examination
Infective endocarditis	Normal echocardiography, blood cultures showed no growth
Tuberculosis	Normal chest X-ray, no granulomatous changes in FNAC
Brucellosis	Negative brucella antibodies
Malignancies	
Acute lymphoblastic leukemia	Normal peripheral film and retics count
Hodgkins/Nonhodgkins lymphoma	No particular findings in FNAC
Autoimmune	
SLE, dermatomyositis, polymyositis	Negative ANA
Post-streptococcal arthritis	Negative RA factor

With the common causes being ruled out, we started to think of AOSD. Serum ferritin and c-reactive protein (CRP) were sent as supportive evidence. Interestingly, both came high (5506 ng/ml and 98.4 mg/L), respectively. Thus, our history, clinical findings, and investigations fulfilled Yamaguchi criteria for adult-onset Still's disease.

## Discussion

Adult-onset Still's disease, first described in children by George Still in 1896, is an uncommon systemic autoinflammatory disease [4]. Its clinical manifestations are broad-spectrum and vague, making the disease go undiagnosed. Different sets of diagnostic criteria have been approved, with Yamaguchi et al. being most sensitive (96.3%) and specific (98.2%) (Table 2) [5].

**Table 2:** Yamaguchi criteria depicting common diagnostic criteria to help guide the diagnosis WBC: white blood cell

Major criteria	Minor criteria	Exclusion criteria
Fever >102F for >1week	Sore throat	Infection
Arthralgia for >2weeks	Lymphadenopathy	Malignancy
Typical rash	Hepatomegaly/splenomegaly	Other rheumatic diseases
WBC >10,000/ml	Abnormal liver function tests	
	Negative antinuclear antibody and rheumatoid factor	

Major criteria include fever (>39°C), joint pains, transient maculopapular rash mostly visible on the chest during a fever spike, and neutrophilic leukocytosis. Minor criteria consist of sore throat, lymphadenopathy/splenomegaly, deranged liver function tests (aspartate aminotransferase (AST), alkaline phosphatase (ALP), alanine transaminase (ALT)), and negative RA and ANA levels. Diagnosis requires at least five criteria consisting of two major and no exclusion criteria. Classification criteria are useful for research, but it lacks the sensitivity and specificity necessary for clinical diagnosis, although the Yamaguchi criteria have the highest sensitivity for diagnosis.

Pathogenesis is quite uncertain. Both genetic factors and a variety of infectious triggers have been suggested to be important, however, there has been no evidence of an infectious etiology. It is not known whether all patients share the same etiopathogenic factors. The most widely accepted mechanism is auto-inflammation, in which a triggering factor(s) stimulate immune response causing the release of pro-inflammatory cytokines leading to systemic inflammation [2,6]. Laboratory tests supporting the diagnosis of AOSD include anemia, leukocytosis, raised lactate dehydrogenase (LDH), ESR, CRP, and ALP [7]. Hyperferritinemia is a characteristic feature of this disease and has been suggested as a serological marker to monitor response to treatment [4,8].

Clinically disease course may be self-limited or monophasic, intermittent or polycyclic systemic, or chronic articular [9]. The former two patterns are more common than the latter [5]. Our patient suffered from monophasic type. The treatment regimen includes corticosteroids as the first line and disease-modifying antirheumatic drugs (DMARDs) reserved as second-line therapy for patients who fail to achieve full remission on corticosteroids, when arthritis is the predominant manifestation, or for those who have evidence of erosive joint disease on hand radiography [10-11]. The patient was started with steroids, which were tapered over a period of one month, and went into remission.

## Conclusions

Adult-onset Still's disease is an uncommon disorder, frequently misdiagnosed due to its overlapping signs and symptoms. It should always be considered in the differential diagnosis for pyrexia of unknown origin. Along with a basic workup of such patients, to rule out common causes of fever, serum ferritin levels should always be sent, which is an important diagnostic marker of adult-onset Still's disease. Serum ferritin concentrations of more than 3,000 ng/ml and sometimes values ​​greater than 10,000 ng/ml have been observed. This will not only avoid unnecessary workup but will also help initiate prompt treatment in the form of steroids or biological inhibitors.
